# A challenging case of an adolescent and young adult patient with high-risk acute lymphoblastic leukemia: the need for a multidisciplinary approach: a case report

**DOI:** 10.1186/s13256-022-03366-y

**Published:** 2022-04-11

**Authors:** Izabela Kranjčec, Nuša Matijašić, Slaven Abdović, Iva Hižar Gašpar, Lavinia La Grasta Sabolić, Filip Jadrijević-Cvrlje

**Affiliations:** 1 Department of Oncology and Hematology, Children’s Hospital Zagreb, Klaićeva 16, 10000 Zagreb, Croatia; 2 Division of Nephrology, Department of Pediatrics, Children’s Hospital Zagreb, Zagreb, Croatia; 3 Department of Pediatrics, Children’s Hospital Zagreb, Zagreb, Croatia; 4 Department of Pediatric Endocrinology, Diabetes and Metabolism, University Hospital Center Sestre milosrdnice, Zagreb, Croatia

**Keywords:** Acute lymphoid leukemia, Hyperglycemia, Chronic kidney diseases, Iron overload, Psychosis, Case report

## Abstract

**Background:**

Adolescents and young adults diagnosed with acute lymphoblastic leukemia are treated according to pediatric-based regimens to achieve better results. However, implementation of intensive chemotherapy protocols in this age group is associated with increased treatment-related toxicities, affecting almost every organ and system. In this case, the focus of our interest was on rather rare entities: steroid-induced psychosis that seldom develops in children and adolescents, and choroid plexus hemosiderosis, infrequently identified as a first sign of iron overload.

**Case presentation:**

The aim of this paper is to present a challenging case of a 15-year-old Caucasian male patient treated for high-risk acute lymphoblastic leukemia and who experienced various adverse incidents during intensive chemotherapy, thus necessitating a high-quality multidisciplinary approach. Slow minimal residual disease clearance was an additional concerning issue. Induction and re-induction were complicated by steroid-induced hyperglycemia that required multiple-week insulin. During consolidation, acute kidney injury on the basis of chronic kidney disease was verified, demanding subsequent drug dose modifications. By the end of re-induction, after dexamethasone cessation, infrequent steroid-induced psychosis, presented as incoherent speech, aggressive behavior, and mood swings, required intensive psychiatric support. Neurological evaluation of seizures revealed uncommon choroid plexus hemosiderosis by brain magnetic resonance imaging, warranting appropriate selection of iron chelation therapy in the context of preexisting nephropathy. Ultimately, iron deposits of moderate intensity were verified by liver magnetic resonance imaging, while heart tissue remained intact. The early diagnosis and adequate treatment of aforementioned difficult toxicities resulted in complete recovery of the patient.

**Conclusions:**

Treating adolescents with high-risk acute leukemia and multiple therapy-related morbidities remains a challenge, even in the era of extensive and effective supportive therapy. Superior survival rates might be achieved by prompt recognition of both frequent and rarely encountered adverse episodes, as well as well-timed and appropriate management by a well-coordinated multidisciplinary team.

## Background

Adolescents and young adults (AYA) diagnosed with acute lymphoblastic leukemia (ALL) have faced poorer survival rates compared with the history of this illness treatment in children [1]. However, several European and US studies have reported improved outcomes for AYA patients treated with pediatric-based protocols [2–4]. however, AYA patients receiving pediatric regimens and doses, unlike children, have disproportionately increased toxicities affecting almost every organ and system [5], most likely due to pubertal changes, inadequate nutritional status, and altered drug metabolism [6]. The most common nonhematological toxicities in AYA patients during induction include hyperglycemia, febrile neutropenia, and transaminitis [3].

The aim of this paper is to present the case of an adolescent with high-risk ALL who experienced various adverse episodes throughout the intensive chemotherapy, including multiple frequent toxicities mentioned above. However, the focus of our interest is on rather rare entities, such as steroid-induced psychosis that seldom develops in children and adolescents, and choroid plexus hemosiderosis, infrequently identified as a first sign of iron overload.

## Case report

A 15-year-old Caucasian male presented with painless cervical lymphadenopathy and excessive sweating. The patient’s family and psychosocial history was unremarkable. Moreover, no relevant past interventions were recorded in the adolescent’s medical history. Normocytic anemia (Hemoglobin 86 g/L Mean corpuscular volume 93.6fL), thrombocytopenia (Plt 49 × 10^9^/L), and blasts (36%) dominated in the peripheral blood. Bone marrow analysis by flow cytometry revealed the diagnosis of precursor B-ALL (60% of aberrant “common” B-cells by European Group for Immunological Classification of Leukemias (EGIL) classification; TdT+, CD19+, CD10+, CD79a+, citIgM−). A favorable hyperdiploid clone (55, XY,  X, +4, +6, +10, +14, +17, +18, +21, +21/46, XY) was detected by classical cytogenetic technique (G-banding). *PBX1* gene duplication and tetrasomy of chromosome 21 were verified by fluorescence in situ hybridization (FISH). Clonal IgH and T-cell receptor (TCR) gene rearrangements were confirmed by molecular analysis (real-time polymerase chain reaction). No unfavorable cytogenetic or molecular disease features (for example, *bcr/abl*, *KTM2A*) were discovered. Additionally, next-generation sequencing (NGS) investigation of the tumor DNA revealed *NRAS* and *CBL* mutations but without potential therapeutic implications. No leukocytes or blasts were discovered in cerebrospinal fluid, and initial brain magnetic resonance (MR) was normal, thus central nervous system (CNS) was free of disease (CNS1 status). Diagnostic assessment was carried out according to the protocol’s standards, and no special (for example, financial) work-up or therapeutic challenges were encountered.

Chemotherapy according to the ALL-Intercontinental Berlin–Frankfurt–Münster (IC BFM) 2009 protocol was initiated, consisting of induction (prednisone, vincristine, daunorubicin, PEG-asparaginase, intrathecal methotrexate), early intensification (cyclophosphamide, cytarabine, 6-mercaptopurine, intrathecal methotrexate), consolidation (combination of dexamethasone, vincristine, vindensine HD-cytarabine, HD-methotrexate, cyclophosphamide, ifosfamide, PEG-asparaginase, etoposide, intrathecal therapy), and re-induction therapy (dexamethasone, vincristine, doxorubicin, PEG-asparaginase, cyclophosphamide, cytarabine, 6-thioguanine), followed by maintenance (6-mercaptopurine, methotrexate). While good prednisone response (peripheral absolute blast count < 1000/µL) was achieved by day 8 (peripheral absolute blast count 237/µL), flow cytometry minimal residual disease (FC-MRD) on day 15 and 33 was 28.9% and 0.03%, respectively. Solely due to high FC-MRD percentage of blasts (> 10%) on day 15, the patient was classified into high-risk (HR) disease group. Persistent minimal residual disease (MRD) (0.0012%) was detected by day 78, no MRD (0%) status was achieved prior to second high-risk block (consolidation), and the patient remained disease-free through further intensive chemotherapy course. Following the decision of the national transplantation team, the patient was not eligible for allogeneic hematopoietic transplantation.

Throughout the 10-month intensive chemotherapy, the patient experienced multiple toxicities of various degrees. Treatment-related adverse events of moderate to higher grade, according to the Common Terminology Criteria for Adverse Events (CTCAE) v4.03, are listed in Table 1. The most troublesome complications warranting multidisciplinary approach are described in more detail below.

**Table 1:** Treatment-related toxicities (common terminology criteria for adverse events grade 2–4) during intensive chemotherapy treatment (Acute Lymphoblastic Leukemia Intercontinental Berlin–Frankfurt–Münster 2009)

Adverse event	Treatment phase	AE grade	Note
Infections and infestations			
Sepsis	Consolidation	4	Citrobacter
Invasive fungal infections	None	N/A	
Blood and lymphatic system disorders			
Febrile neutropenia	Induction, consolidation, re-induction	3	
Anemia	Induction, consolidation, re-induction	3	
Platelet count decreased	Induction, consolidation, re-induction	3–4	
Coagulation disorders (fibrinogen decreased)	Induction, consolidation, re-induction	2–3	
Gastrointestinal disorders			
Mucositis	Induction, consolidation	3	
Pancreatitis	None	N/A	
Hepatobiliary disorders			
Transaminitis (ALT, AST increased)	Consolidation, re-induction	3	
Blood bilirubin increased	Early intensification	2	
GGT increased	Early intensification, consolidation, re-induction	3 3–4	
Metabolism and nutrition disorders			
Hyperglycemia	Induction, consolidation, re-induction	3	Steroid-induced
Anorexia (malnutrition)	Induction, consolidation	2–3	Clinically grade 2, laboratory and neuroradiology results indicate a higher grade
Iron overload	Re-induction	2	
Endocrine disorders			
Adrenal insufficiency	Induction, consolidation, re-induction	2–3	
Hypothyroidism	Induction	2	
Vascular disorders			
Hypertension	Induction, ongoing	3	
Thromboembolic events	None	N/A	
Renal and urinary disorders			
Chronic kidney disease	Consolidation	2	Underlying condition
Acute kidney disease	Early intensification	3	Amfotericin B-induced
Nervous system disorders			
Polineuropathy	Induction, ongoing	3	
Convulsions	Re-induction	2	
Psychiatric disorders			
Psychosis	Re-induction	3–4	Steroid-induced
Immune system disorders			
Allergic reactions	None	N/A	
Musculoskeletal and connective tissue disorders			
Avascular necrosis	None	N/A	
Osteoporosis	Re-induction	2	
Skin and subcutaneous tissue disorders			
Dermatitis	Re-induction	2–3	Steroid-induced

*CTCAE* common terminology criteria for adverse events; *N/A* non applicable; *ALT* alanine aminotransferase; *AST* aspartate aminotransferase; *GGT* gamma-glutamyl transferase; *AE* Adverse event

### Endocrine system

Hyperglycemia (serum glucose 12.1 mmol/L) was first noted on the third day of induction, during increase of prednisone dose (beginning with 25% of the calculated dose, 25% daily increments, full dose of 60 mg/m^2^/day reached on the 4th day), when nutritional advice was sought. As serum glucose levels (17.3 mmol/L) had risen additionally by day 9, intensive insulin therapy was initiated, intermittent-scanning continuous glucose monitoring system was applied, and education of the patient and his mother was performed in detail. Further workup (Glycated hemoglobin, C-peptide, diabetes related autoantibodies) excluded the possibility of type 1 diabetes, and hyperglycemia had resolved after 31 days of insulin therapy. Short-term steroid use (dexamethasone 20 mg/m^2^/day over 5 days) during consolidation demanded solely nutritional adjustments. During the re-induction, hyperglycemia (serum glucose 14.5 mmol/L) appeared the third day of dexamethasone use (10 mg/m^2^/day), necessitating multiple daily insulin injections (basal-bolus regimen) for 34 days. Adrenal insufficiency (cortisol 63 nmol/L) and central hypothyroidism (triiodothyronine < 0.80 nmol/L, thyroxine 47 nmol/L, thyroid stimulating hormone 0.04 mU/L) were detected by the end of the fourth week of induction and third week of steroid therapy in re-induction, warranting 2-week hormone replacement therapy (levothyroxine, hydrocortisone) with prolonged tapering.

### Renal system

**Fig. 1:**
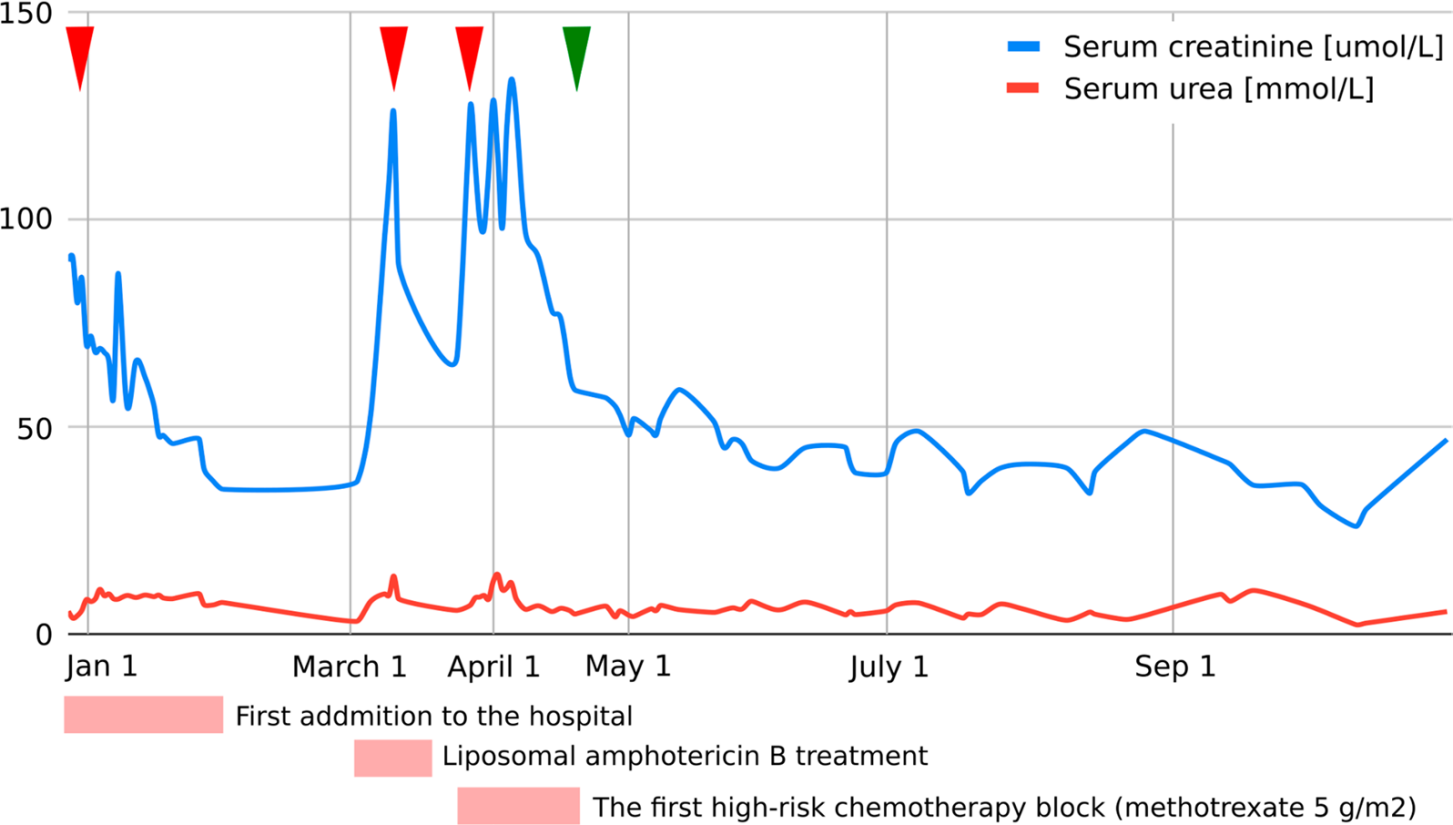
Graphic presentation of the renal function (serum creatinine, serum urea) during intensive treatment. First acute kidney injury was diagnosed when the patient was initially admitted to the hospital with creatinine levels of 90 µmol/L, which decreased to reference values for age with an intensive rehydration regimen (left arrowhead). At that time, kidney morphology was evaluated with ultrasound, which showed normal dimensions and echomorphology without dilatation of the urinary tract. Second (middle arrowhead) and third episodes (right arrowhead) were classified as acute kidney failure and occurred during liposomal amphotericin B treatment and during the first high-risk chemotherapy block when a significant delay (198-hour) in high-dose methotrexate (5 g/m^2^) metabolite excretion was noticed, resulting in transient rise of creatinine and cystatin C levels up to 125 µmol/L and 2.36 g/L, respectively (estimated Glomerula Filtration Rate 30 mL/min/1.73 m^2^). Creatinine levels returned to normal when replacing amphotericin B with voriconazole and monitoring complete methotrexate elimination. Apart from urine alkalinization, increased hydration, and administration of leucovorin, no other treatments were necessary to resolve acute kidney injury. Renal Tc-99m Diethyl Triamine Penta-Acetic scintigraphy scan revealed decreased clearance of radiopharmaceutical material (75 mL/min/1.73 m^2^), and chronic kidney disease grade 2 was diagnosed. The patient had previously (at age of 3) been followed by pediatric nephrologist due to congenital hydronephrosis, but renal function and morphology were reported normal. We presume the patient initially had reduced renal parenchymal reserve and was more prone to acute kidney injury during precipitating factors (dehydration and unadjusted drug doses). Further cytostatic and symptomatic therapy dose corrections (75% of the total methotrexate dose and avoidance of all nephrotoxic drugs) were consistently undertaken, and laboratory parameters carefully monitored (starting from green arrowhead), so no additional kidney function deterioration was observed

As evaluated by Pediatric Risk, Injury, Failure, Loss, End Stage Renal Disease (pRIFLE) criteria [7], our patient developed acute kidney injury (AKI) on three occasions, details of which are presented in Fig. 1.

### Nervous system and psychological status

One day after a 3-week dexamethasone course (10 mg/m^2^/day) with 1-week tapering, at the end of the first part of re-induction, a bizarre behavior pattern was observed. The patient’s speech was incoherent, and aggressive outbursts were replaced by manic-depressive mood swings. A sudden-onset qualitative consciousness disturbance accompanied by short tonic–clonic convulsions demanded prompt neurological evaluation. No electrolyte disorders, abnormal glucose levels, or high blood pressure readings were detected. Urine toxicological screening was negative. Urgent head computed tomography (CT) scan was unremarkable, and repeated electroencephalogram (EEG) recordings were normal. Magnetic resonance imaging (MRI) of the brain revealed choroid plexus hemosiderosis, providing no explanation for psychological status alteration. Polymerase chain reaction (PCR) encephalitis and meningitis panels (Borrelia, tick-borne encephalitis, herpes simplex virus type 1 and type 2, varicella-zoster virus, cytomegalovirus, Epstein-Barr virus, measles, mumps, Human herpesvirus 6) in cerebrospinal fluid were negative, as were autoimmune encephalitis autoantibodies. A child psychiatrist diagnosed the patient with steroid-induced psychosis and introduced medications (risperidone, promazine), along with intensive psychological support. Within a week, all symptoms ceased, and the patient’s psychological status remained stable even after the drugs’ discontinuation. No new cerebral events were described, and follow-up EEG and neurological status were satisfactory.

### Iron overload

**Fig. 2:**
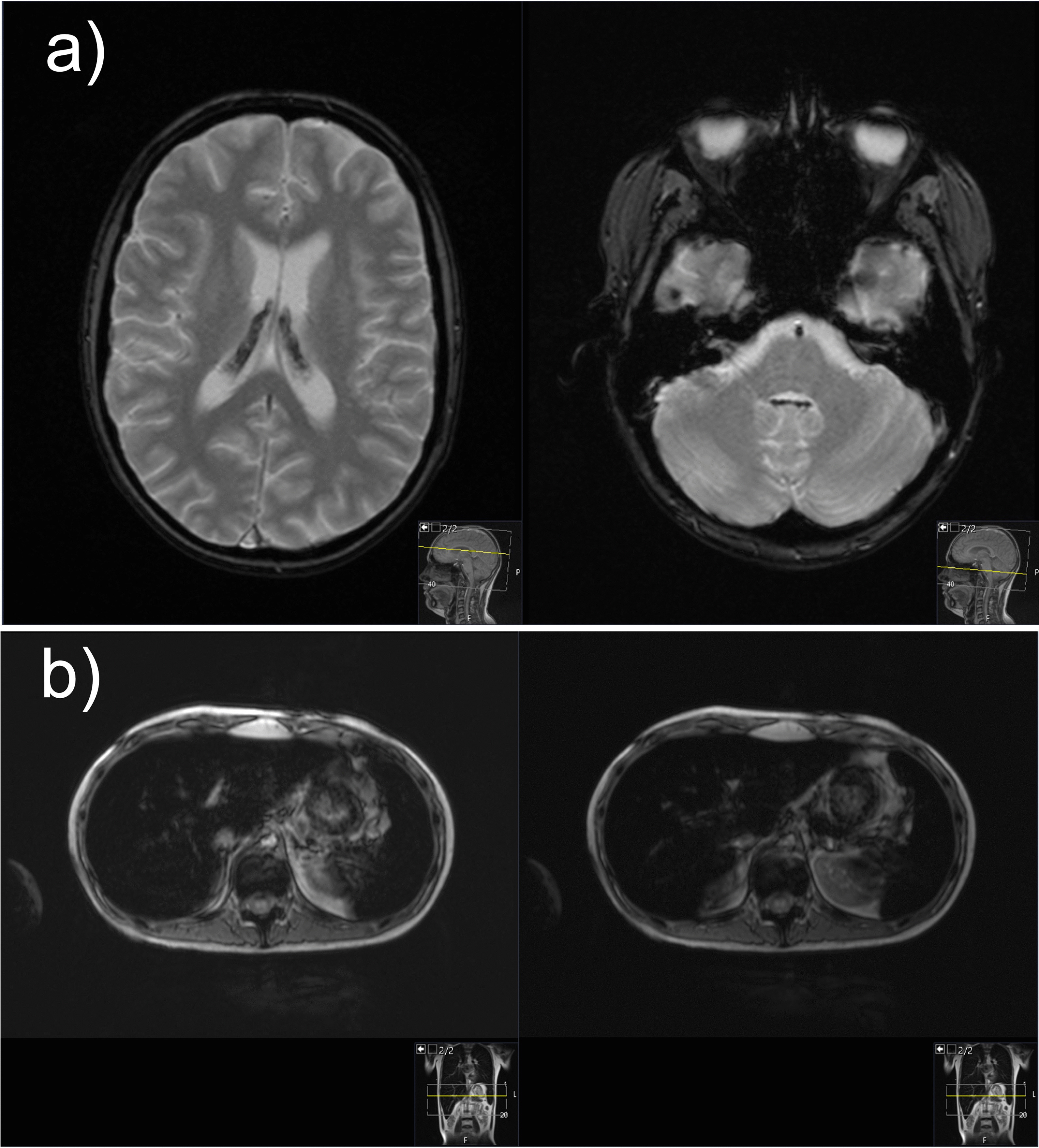
Magnetic resonance images of the brain and liver demonstrating iron overload. **a** Magnetic resonance imaging of the brain: axial T2-weighted gradient echo images demonstrate presence of hypointense hemosiderin deposits in the choroid plexus of both the lateral ventricles and fourth ventricle. **b** Magnetic resonance imaging of the liver: axial gradient echo sequences T2-weighted magnetic resonance image shows the liver hypointensity that is due to iron overload

Hemosiderosis, due to repeated blood transfusion, was revealed during MRI of the brain and confirmed with high ferritin levels and MRI of the liver (Fig. 2). The baseline ferritin level of 323 µg/L (serum iron 20 µmol/L, total iron-binding capacity 43 µmol/L, unsaturated iron-binding capacity 23 µmol/L) in our patient had risen to 5143 µg/L after 9 months of intensive chemotherapy and then spontaneously fallen to 2994 µg/L at 1 month after (ferritin reference range 10.3–55.8 µg/L). During intensive chemotherapy, the patient received a total of 43 doses of blood (red cell) transfusions. After only a month of chelation therapy (deferasirox 20 mg/kg/day), a significant decrease to 1664 µg/L was noted with no deterioration in kidney function observed.

## Discussion and conclusions

B-precursor HR-ALL AYA patients are known to have inferior outcomes and increased treatment-related toxicities compared with children [8]. Superior outcomes in AYA patients are, however, achieved by implementing more intensive, pediatric-type protocols, with survival rates reaching 70% [9]. Lower event-free and survival rates in AYA patients are, among other factors, due to unfavorable tumor biology. The frequency of Philadelphia Chromosome positive acute lymphoblastic leukemia (Ph+ALL) and other HR abnormalities increases with age, in contrast to favorable cytogenetics in younger patients [9]. Although our patient displayed no disadvantageous genetic features, persistent MRD, known to be of extreme prognostic relevance, raised great concern. While MRD clearance, although slow, was still achieved in consolidation and allogeneic transplant avoided, a multitude of newly arising toxicities remained a challenge.

One in ten pediatric patients experiences hyperglycemia as a common side effect of ALL treatment [6]. Grade 3–4 hyperglycemia, a major concern in our patient throughout the treatment, was observed in almost 30% of AYA population treated with Children's Oncology Group (COG) pediatric-inspired chemotherapy regimens [10]. Higher proportion of steroid and asparaginase-related hyperglycemia in AYA patients might be the result of postpubertal hormonal changes, and given the former contradictory results, the correlation with infection predilection remains to be determined [6].

Although the frequency of febrile neutropenia (FN) in AYA patients is reported lower compared with younger individuals, our patient developed fever during hematologic aplasia in every stage of intensive chemotherapy. However, an association to hyperglycemia was not apparent [8]. Nevertheless, malnutrition has been repeatedly described as a predisposing factor for FN [11–13].

Although less than 10% of AYA patients are malnourished at time of diagnosis, almost half of them experience more than 5% weight loss during cancer treatment [14]. Not only was our patient severely underweight (Body mass index, BMI 15.9 kg/m^2^) when first faced with hematologic malignancy, but significant weight loss (14%) was also observed during induction. High-risk disease and hyperglycemia, both present in our patient, among other features, are recognized as risk factors for negative weight trend during induction [15]. However, an early dietitian referral and specialist gastroenterologist involvement, along with timely enteral nutrition and supplement introduction, resulted in desirable weight gain at the end of intensive chemotherapy regimen (+4 kg).

Hepatotoxicity, sometimes severe, is a common side effect of contemporary pediatric ALL regimens, in one-fourth of cases occurring during induction, with obesity and age (> 15 years) being regarded as risk factors [16]. Transitory rise in transaminases in our patient followed every administration and was related to PEG-asparaginase exposure but never required any specific interventions nor treatment postponement (max ALT 342 U/L, AST 265 U/L, GGT 482 U/L). Other PEG-asparaginase attributable toxicities, such as hypersensitivity reactions, pancreatitis, and thromboembolic events, were not described in our patient [17]. However, therapeutic drug monitoring (TDM) was performed and dose modification conducted, possibly decreasing the occurrence of asparaginase-related adverse events.

Increased risk of high-dose methotrexate (MTX) renal toxicity was found to be correlated with delayed MTX elimination [18, 19]. Since serum creatinine levels and concentration of MTX after 48 hours had excellent value in predicting AKI, with area under the curve (AUC) of 89.2% and 96.8%, respectively, it is necessary to follow-up these values and initiate intravenous hydration, urine alkalization, and if necessary, renal replacement therapy on time.

Patients with HR-ALL are at higher risk for iron overload, accompanied by endocrine and liver dysfunction, compared with other risk groups, yet no regular iron status monitoring is routinely performed in many pediatric oncology centers [20, 21], a practice that our center should consider implementing. Accumulation of iron in the liver correlates with the amount of transfused iron, occurring rather early, after as much as ten transfusions, while for the iron to be loaded in the heart and endocrine organs, high transferrin saturations are needed [22]. Moreover, choroid plexus iron depositions, at any stage of treatment, have rarely been described in literature [23]. In our patient, the peak ferritin level correlated with neurological and psychological deterioration and characteristic brain MRI findings of iron overload (Fig. 2). Iron deposits of moderate intensity were verified by liver MRI, while heart tissue remained intact. Hereditary hemochromatosis gene (HFE) mutations, which aggravate iron overload in ALL patients, were not detected in our case [24]. Hepatic dysfunction, dysglycemia, and endocrine disorders, such as primary thyroid gland hypofunction, are organ abnormalities commonly related to hemosiderosis in literature [25]. Occasional hepatotoxicity, central hypothyroidism, and hypocortisolism in our patient were not considered to be related to hemosiderosis, as explained above, but preexisting nephropathy played an important role in iron chelation selection. Orally active once-daily deferasirox is a frequently preferred iron chelator, especially in the outpatient setting [25, 26], usually being well tolerated [27], as in our case. Nevertheless, regarding clinical presentation, neurological symptomatology could not be interpreted based on choroid plexus hemochromatosis, as it is mostly asymptomatic [23], so further elucidation was sought.

Steroid-induced psychosis, a variety of neuropsychiatric symptoms related to glucocorticoid use, is frequently described in adult populations rather than children, with fewer than 20 cases reported worldwide [28]. All routes of corticosteroid administration (oral, intravenous, inhalation) at any time point of treatment may provoke psychotic symptoms, but clear risk factors (for example, type and dose of glucocorticoid) have not yet been established [29]. Hippocampal injury caused by glucocorticoids, resulting in behavioral and emotional dysfunction, in pediatric patients with cancer might be aggravated by synergistic toxicity of other chemotherapeutic agents [30]. The hallucinations that arose in our patient with negative personal and family history of psychiatric disorders and that caused him significant stress and impairment were associated with recent steroid exposure, while infection, severe electrolyte, and serum glucose disorders were ruled out. Combination of an antipsychotic drug (for example, haloperidol) with steroid dose reduction or discontinuation is generally an effective treatment strategy [31]. Risperidone, a preferred antipsychotic in literature [28, 32], was also our child psychiatrist’s medication of choice, leading to complete symptom resolution. An optimal medicamental prophylactic approach (for example, carbamazepine, chlorpromazine, etc.) still needs to be determined [31].

In conclusion, treating an AYA patient with high-risk leukemia and multiple therapy-related morbidities remains a challenge, even in the era of abundant and effective supportive treatment. A timely and appropriate multidisciplinary approach is mandatory to ensure no significant delays, and modification in scheduled therapy is required, late effects diminished, and quality of life preserved, to achieve optimal treatment outcomes.

## Data Availability

The datasets used and/or analyzed during the current study are available from the corresponding author on reasonable request.

## Abbreviations

AKI: Acute kidney injury
ALL: Acute lymphoblastic leukemia
ALL-IC BFM: Acute Lymphoblastic Leukemia Intercontinental Berlin–Frankfurt–Münster
ALT: Alanine aminotransferase
AST: Aspartate aminotransferase
AYA: Adolescents and young adults
BMI: Body mass index
CNS: Central nervous system
COG: Children's Oncology Group
CT: Computed tomography
CTCAE: Common Terminology Criteria for Adverse Events
DTPA: Diethyl Triamine Penta-Acetic scintigraphy
EEG: Electroencephalogram
e-GFR: Estimated glomerula filtration rate
EGIL: European Group for Immunological Classification of Leukemias
FISH: Fluorescence in situ hybridization
GGT: Gamma-glutamyl transferase
GRE: Gradient echo sequences
FN: Febrile neutropenia
FC-MRD: Flow cytometry minimal residual disease
HFE: Hereditary hemochromatosis
HR: High risk
MR: Magnetic resonance
MRD: Minimal residual disease
MRI: Magnetic resonance imaging
MTX: Methotrexate
PCR: Polymerase chain reaction
Ph+ALL: Philadelphia Chromosome positive acute lymphoblastic leukemia
pRIFLE: Pediatric Risk, Injury, Failure, Loss, End Stage Renal Disease
TDM: Therapeutic drug monitoring
